# The Western News Company

**Published:** 1869-05-01

**Authors:** 


### *The Western News Company.*

A few days since was opened in Chicago one of the most extensive and costly-fitted-up Book Stores in the United States. We refer to the Western News Company, 121 and 123 State street, John R. Walsh, Manager. The new quarters of this enterprising company embrace two store fronts. On entering, one is impressed with the richness and beauty of the appointments of this spacious store.

On either side are ranged beautifully carved and adorned book-cases of walnut and chestnut. The style is the pointed or early Gothic, surmounted by pinnacles, the arrangement being so artistic and consummate as to render the whole a series of complete book-cases, of construction and style as elaborate and expensive as any to be found in the private library of the wealhiest book lover. Each set of cases is surmounted by a hooded cornice of walnut, relieved by a groundwork of chestnut, with Gothic mouldings, open quartrefoil tracery above, and a pointed pinnacle surmounting each division. In front of each pilaster is placed a solid walnut column finished with carved capitals. Midway of the store on each side, is placed a gothic niche which forms a perfect division or line of demarkation between the elaborate ornamentation which prevails in the front half, the retail portion of the establishment, and the plainer, though exquisitely beautiful style which finds appropriate place iu the wholesale department. These dividing niches stand out in relief past the cases, each being tipped with a pointed gothic hood supported by four walnut columns with carved capitals, and resting upon solid octagon pedestals. The hood is surmounted by carved finial, with open quatrefoil tracery on the ridge.

We have not space to enter into the details of the various departments, but all are arranged in fine taste and convenient for the transaction of business.

In connection with books, rich and rare, from all quarters, an immense trade is done in newspapers and periodlcals, with news dealers all over the country.

Mr. John R. Walsh, in fifteen years in Chicago, has risen from obscurity to be the manager of one of the most extensive businesses in the country. We invite all to call and see his beautiful store.

